# A Comparative Evaluation of Soft and Hard Tissue Changes Around Dental Implants Placed With and Without Platelet-Rich Fibrin

**DOI:** 10.7759/cureus.36908

**Published:** 2023-03-30

**Authors:** Kanchan Sharma, Suparna Roy, Archana Kumari, Marupaka Bhargavi, Sumati Patel, Prasad Ingale, Rashmi Laddha

**Affiliations:** 1 Department of Orthodontics and Dentofacial Orthopaedics, Awadh Dental College and Hospital, Jamshedpur, IND; 2 Department of Prosthodontics and Crown & Bridge, Awadh Dental College and Hospital, Jamshedpur, IND; 3 Department of Oral and Maxillofacial Surgery, Panineeya Institute of Dental Sciences and Research Centre, Hyderabad, IND; 4 Department of Periodontology, Babu Banarasi Das College of Dental Sciences, Lucknow, IND; 5 Department of Conservative Dentistry and Endodontics, Bharati Vidyapeeth Dental College and Hospital, Sangli, IND; 6 Department of Periodontology, Dr. Rajesh Ramdasji Kambe Dental College and Hospital, Akola, IND

**Keywords:** periosteal reaction formation, platelet rich fibrin, soft tissue seal, crestal bone loss, dental implants

## Abstract

Background: A patient's ability to maintain a healthy bone-implant interface seems to be a major predictor of implant longevity over the long term. The implant surface is protected from the oral environment, the bone, and the implant itself by the peri-implant tissues. Platelet-rich fibrin (PRF) has been shown to help in the regeneration of bone and other connective tissues. Since there has been inadequate information on the role of PRF in maintaining soft tissue integrity and crestal bone changes, the present study aimed to evaluate these challenges clinically and radiographically in human patients who had dental implants placed with PRF.

Materials and methods: There were a total of 15 patients who were recalled for the analysis, and they were split into two groups. PRF was used to complete the implant procedure in the experimental group, but PRF was not used in the control group. Cone beam computed tomography (CBCT) was used to evaluate the amount of alveolar bone prior to dental implant placement and intra-oral periapical radiograph (IOPAR) for postoperative assessment. Gingival index, plaque index, probing depths, papilla bleeding index, and crestal bone changes were used to document clinical limits. IOPAR using a similar approach was used to evaluate the crestal bone level alterations. Patients were evaluated clinically and radiographically for changes in the peri-implant soft tissue and crestal bone during implant placement, six and nine months postoperatively.

Results: From baseline (p=0.02) to six months (p=0.04) and nine months (p=0.04), both groups showed changes in crestal bone loss and soft tissue although the changes in the test group were smaller. Soft tissue changes showed significant differences for probing depth and papilla index score at baseline and at the end of the six and nine months (p<0.05), whereas no significant difference was noted with bleeding index and plaque index score during the follow-up (p>0.05).

Conclusion: To conclude, the provided data demonstrated that the local injection of PRF during implant placement has the potential to favorably stimulate bone formation, and may be used as a therapeutic adjuvant in the clinical setting of implant placement.

## Introduction

Oral rehabilitation, a subspecialty of contemporary dentistry, makes substantial use of medical data throughout the diagnostics, treatment planning, repair of dental defects, and replacement of missing teeth processes (whether they are acquired or congenitally present) [1]. Modern implant-supported prostheses for replacing lost teeth are considered a standard practice in the field of dentistry, restoring patients' oral health and improving their appearance [2]. Over the last decade, dental implants have shown to be an effective method for restoring teeth in patients who are missing some or all of their natural teeth [3]. Successful healing and integration of implant components with hard and soft oral tissues are crucial to the long-term success of dental implants [4].

In order to get a desirable aesthetic result, it is necessary to recreate the volume, color, and form of natural, healthy supra-implant gingiva. The shape of the soft tissue that sits above an implant is heavily influenced by the properties of the bone and soft tissue around the implant as well as the implant restorations themselves. Implants that blend in with the adjacent natural teeth need careful consideration of the amount of interproximal soft tissue and the positioning of the mid-facial gingiva [5]. Implants are considered successful if they are immobile, free of peri-implant radiolucency, free of infection in the peri-implant soft tissue, have an acceptable breadth of connected gingiva, and have little crestal bone loss [6].

At the site of soft and hard tissue damage, blood platelets produce platelet-derived growth factor (PDGF) during clotting, which then triggers a series of events leading to a wound-healing response. Using PDGF-BB to treat periodontal wounds has been shown to speed up the healing process and promote substantial bone, cementum, and periodontal regeneration. These findings demonstrate the positive impact of PDGF on tissue regeneration and repair in both soft and hard tissues [7]. Due to recent developments in tissue engineering, growth factors are being used to speed up the osseointegration process. In order to realize the potential of bioactive protein coatings for dental implants, Hall used a titanium porous oxide oral implant surface as a carrier for recombinant human bone morphogenetic protein 2 (rhBMP-2) and rhBMP-7 [8].

When platelets, growth factors, and leukocyte cytokines are enriched into fibrin membranes, the resulting platelet-rich fibrin (PRF) is utilized to accelerate the healing of soft and hard tissues. PRF was initially identified by Choukroun and coworkers in France in 2001 [9]. PRF's greatest strength is its ability to expedite the healing process by depositing a highly concentrated mixture of growth factors (such as transforming growth factor beta-1, PDGF-AB, vascular endothelial growth factor, or VEGF) and matrix proteins (such as thrombospondin-1, fibronectin, vitronectin) at the surgical site. Strongly promoting angiogenesis, immunological regulation, and stem cell mobilization are just a few of the many ways that PRF aids in the repair of both soft and osseous tissues [10]. PRF with a dense fibrin network releases growth factors and glycoproteins gradually over the course of many days. Soft and hard tissue recovery may be accelerated by this bioactive barrier, which also serves to shield surgical sites and grafted materials from harmful environmental factors. Many different cell types were examined in vitro to determine the effects of PRF membranes on proliferation, and it was expected that leukocytes would have an impact on both cell responses and growth factor release [11].

The effectiveness of PRF in improving and maintaining the peri-implant osseous structure and gingival architecture has to be verified. The purpose of this research was to compare the soft tissue and crestal bone alterations radiographically and clinically after dental implant insertion with and without the use of PRF.

## Materials and methods

Patients were recruited from the outpatient department of prosthodontics at Awadh Dental College and Hospital, Jamshedpur, with an approval from the institutional review board (IEC/2020/IMP/11). Patients from the surrounding community who presented with tooth loss were included in the trial. Endosseous implants were used to restore their smiles. Patients in this prospective, randomized, controlled trial ranged in age from 21 to 55 years, and had their implants placed in 20 different locations (mean age, 38 years). Blinding was not performed, and both the patient and the operator were aware of the treatment category to which they were being assigned. The sample size was determined using the mean and standard deviation values from the literature [5]. Sites for implants were randomly assigned to one of the following categories: test group (10 patients) implants placed with platelet-rich fibrin and control group (10 patients) implants placed without platelet-rich fibrin.

Patients' complete medical history was gathered and the following tests were recommended before implant surgery could begin with preoperative assessment by cone beam computed tomography (CBCT) for better result outcomes. A complete hemogram, and random blood sugar level test were also done.

Patients were given information on the procedure's possible advantages and drawbacks, and their written permission was acquired using the standard procedure. The following factors were considered while including patients: good oral hygiene and suitability for implant rehabilitation in a two-stage procedure, sufficient bone volume and bone density (D-2) to receive an implant, stable occlusal relationship, mandibular posterior region implant site free of infection and/or extraction remnants with a history of extraction minimum six months ago. The dimensions of the implant were selected depending on CBCT measurements. Exclusion criteria included the following: a patient not cooperating, any potential risks associated with placing the implant (such as a history of cancer, chronic bone disease, or radiation exposure), a systemic condition present or a patient taking any medicine that should not be taken during implant treatment, presence of any oral parafunctional habits, if the patient was pregnant or lactating and if the patient was a smoker.

A two-stage implant placement procedure was followed. To make PRF, a digital centrifuge machine was used based on the method by Dohan et al. [12]. Ten milliliters of whole venous blood was drawn through venipuncture from the antecubital fossa and placed in anti-coagulant-free sterile vacutainer tubes. After the vacutainer tubes had been loaded, they underwent 10-minute centrifugation at 3000 revolutions per minute. Platelet-rich fibrin was isolated in the resulting intermediate layer. After the PRF was ready, it was compacted into a membrane and cut in half.

Surgical procedure

A single post-graduate trainee performed the surgical procedure for the placement of the implant in both groups.

Control Group

Beneficiaries in the control group got implants devoid of PRF. After assessing the pretreatment records that aided in identifying vital anatomic landmarks, patients were prepared for implant placement. Povidone iodine was used to clean the mouth and teeth, followed with a final rinse of 10 ml of 0.2% chlorhexidine. A local anesthetic (lignocaine 2% with adrenaline) was used to numb the surgical region. A crestal incision was made to expose the alveolar ridge with a No. 12 Bard-Parker blade. Raising a full-thickness mucoperiosteal flap required making a mid-crestal incision on the ridge and sulcular incisions around the neighboring teeth. Prior to the beginning of the drilling technique, the operative site was curetted. The bone was reshaped using a crestotome so that it would be more conducive to receiving an implant (Adin conical shape; Adin Dental Implant Systems, Afula, Israel). The Adin spiral pattern implant may be redirected during implantation with little to no loss in stability. Even in this situation, the dual cutting blade edge design aids in minimizing stress to the osteotomy site, surrounding soft tissue, and bone. A presurgical prosthetic guidance was used to choose the best implant position. A surgical stent was penetrated with a lance drill at a depth matching the implant length that was selected. Following that, a 2-mm punch drill or round bur with a drill speed of 1000 rpm and a torque of 30 N.cm was employed along with extensive internal and exterior sterile saline irrigation. We used a paralleling instrument to double-check the location and orientation of the recipient site. Drilling followed in order to enlarge the site for the implant size chosen. With a speed of 1000 rpm and continuous irrigation, the next bigger implant drill was used to do countersinking (2 mm).

Test Group

Once the test group's osteotomy site was at the appropriate size, PRF was inserted into the site with tweezers before implants were inserted. Using a torque ratchet, the implant was extracted from its sterile container in an aseptic manner. The cover screw was installed using the 0.05″ hex driver. At this point, the implant was immobile, which ensured primary stability. An additional PRF covering was applied to the implant. Once the flap's approximated position was determined, simple interrupted sutures were performed using 3/0 silk (natural silk, India), to seal the incision. The patients were reviewed again after a week. Afterward, sutures were taken out and the wound was examined for any further signs of infection.

The healing period for the implant site was four months. A periapical radiograph was taken prior to the restorative phase and assessed for any peri-implant abnormality adjacent to the implants. The implant was exposed by makking a mid-crestal incision followed by the reflection of the full-thickness flap. A cover screw was removed and replaced by a gingival former. For the test group, PRF was placed with the gingival former such that it covered the peri-implant bone. For both the groups, the gingival former was left for two weeks. Prosthetic procedures were carried out after two weeks following the placement of the gingival former. A gingival cuff or gingival collar formed as a consequence of this. An imprint coping was glued onto the implant after the gingival former was taken away. An impression was taken using a closed tray method as it maintained implant parallelism. To finish off the prosthesis phase, the prosthetic lab created a porcelain crown that was fused to a metal base and it was then affixed to the abutment.

Clinical parameters were assessed by a single evaluator at different time intervals (at baseline, six and nine months after loading) in both test and control groups. The assessment group the evaluator was evaluating was kept hidden from him. The following clinical parameters were recorded at mesial and distal surfaces: bleeding index, plaque index, Jemt papilla index scores, probing depth and crestal bone changes. The quality, quantity, and dimensions of the available bone were evaluated preoperatively using radiographic means, specifically, by means of cone beam computed tomography.

After the first post-loading examination (the baseline), an intra-oral periapical radiograph (IOPAR) with the long-cone paralleling technique was used to conduct follow-up assessments six and nine months later due to expense problems. To facilitate consistent radiographic evaluation, a film holder template was positioned in the same relative position on the neighboring teeth, and an extension arm was used to bring the film and X-ray tube into parallel alignment. The crestal bone height was assessed on the proximal and distal sides of the implants. The distance between the margin of the implant collar and the apex of the patient's jaw was measured. Using an HP X-ray scanner (HP, Palo Alto, CA), we measured the bone height on the proximal and distal sides of the IOPAR. With the UTHSCSA ImageTool, version 3.00 for Windows (University of Texas Health Science Center, San Antonio, TX), precise measurements were taken.

All implants had restorations made out of cement-retained screws and metal-ceramic after four months. Neither implant-related complications (such as implant mobility, implant loss, or abutment screw loosening) nor super-structure-related complications (such as restorative material chipping or fracture) were seen at the subsequent checkup (six and nine months). Crestal bone alterations were evaluated by radiography at three time points (preload, six months postload, and nine months postload) in both groups. Statistics were analyzed using the Student t-test to see whether there were any significant differences in the two groups' assessments of crestal bone loss and soft tissue. The significance level was set at p<0.05. For statistical computations, SPSS, version 21.0, for Windows (IBM Corp., Armonk, NY) was used.

## Results

An intergroup comparison of clinical parameters (plaque index and bleeding index) in test and control groups at baseline, six and nine months using Student's t-test is shown in Tables 1, 2.

**Table 1:** Comparison of bleeding index between test and control groups p<0.05 is significant.

	Test group (mean±SD)	p value	Control group (mean±SD)	p value	p-value of test vs. control
Baseline	30.0±2.22		22.1±8.22		0.13
6 months	29.2±3.22	0.33	21.1±6.34	0.66	0.13
9 months	28.4±4.2	0.45	20.2±5.45	0.44	0.15

**Table 2:** Comparison of plaque index between test and control groups p<0.05 is significant.

	Test group (mean±SD)	p value	Control group (mean±SD)	p value	p value of test vs. control
Baseline	0.34±0.22		0.34±0.22		0.5
6 months	0.36±0.31	0.66	0.28±0.18	0.66	0.5
9 months	0.32±0.21	0.77	0.30±0.34	0.78	0.6

For plaque index and bleeding index, in the test group, there was no statistically significant difference between baseline, six, and nine months. In addition, neither at the beginning nor at the end of the study (nine months) did the test group vary significantly from the control group.

An intergroup comparison of clinical parameters (probing depth) for test and control groups at baseline, six and nine months using Student's t-test is shown in Table 3.

**Table 3:** Comparison of probing depth between test and control groups p<0.05 is significant.

	Test group (mean±SD)	p value	Control group (mean±SD)	p value	p value of test vs. control
Baseline	1.48±0.2		1.8±0.36		0.02
6 months	1.45±0.4	0.83	1.8±0.38	0.94	0.04
9 months	1.38±0.3	0.56	1.8±0.39	0.75	0.04

For probing depth, in the test group, no significant change was seen from the beginning of the study to either six or nine months later. In the control group, no significant change was seen from the beginning of the study to either six or nine months later. At baseline and at both six and nine months, there was a statistically significant difference (p<0.05) between the test and control groups.

An intergroup comparison of clinical parameters (Jemt papilla index score) for test and control groups at baseline, six and nine months is shown in Table 4.

**Table 4:** Comparison of Jemt papilla index changes between test and control groups p<0.05 is significant.

		Test (mean±SD)	p value	Control (mean±SD)	p value	p value of test vs. control
Mesial side	Baseline	2.6±51		1.875±0.353		0.04
	6 months	2.6±51	1	2±0.534	0.54	0.05
	9 months	2.65±0.51	1	2±0.534	0.55	0.03
Distal side	Baseline	2.75±0.46		2±0.755		0.03
	6 months	2.625±0.517	0.65	2±0.534	1	0.03
	9 months	2.37±0.517	0.40	1.8±0.353	0.65	0.04

For Jemt papilla index score, in the test group, there was no statistically significant difference between baseline, six and nine months. In the control group, there was no discernible improvement between the first and second assessment (six months) or the third (nine months) assessment. There was a significant difference between the test and control groups at baseline and at the end of the six and nine months of the review.

A radiographic assessment comparison of crestal bone changes in test and control groups from baseline to six and nine months is shown in Table 5.

**Table 5:** Comparison of crestal bone loss of mesial and distal surfaces in test and control groups p<0.05 is significant.

		Test (mean±SD)	p value	Control (mean±SD)	p value	p value of test vs. control
Medial side	Baseline	0.10±0.05		0.28±0.22		0.02
	6 months	0.11±0.07	0.45	0.42±0.34	0.26	0.01
	9 months	0.17±0.16	0.28	0.55±0.47	0.20	0.04
Distal side	Baseline	0.15±0.11		0.19±0.15		0.04
	6 months	0.08±0.09	0.34	0.32±0.22	0.11	0.03
	9 months	0.11±0.08	0.45	0.34±0.19	0.13	0.01

**Figure 1:**
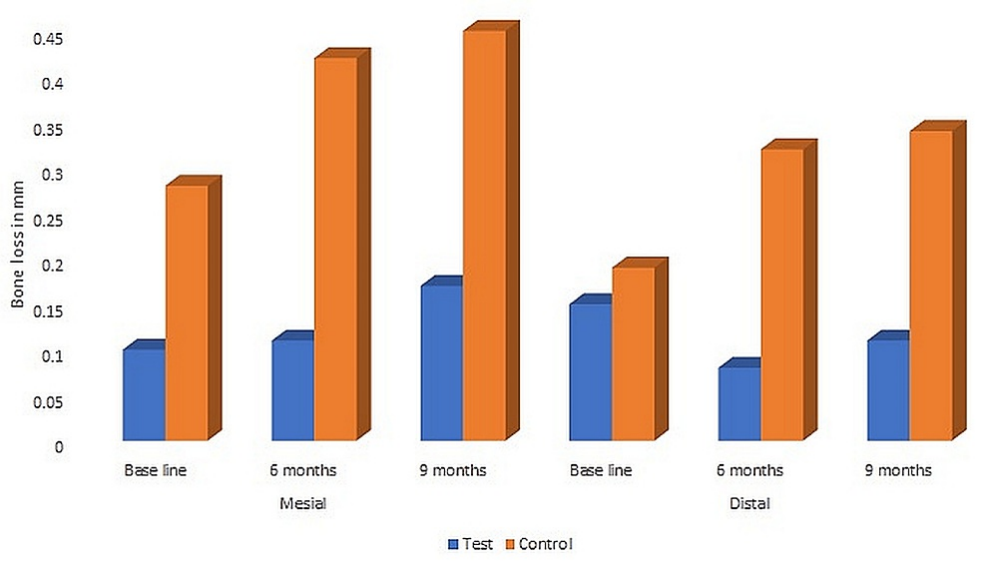
Graph showing a comparison of the crestal bone level changes in test and control groups

Both groups were evaluated for crestal bone level changes at baseline (at the time of loading), six months, and nine months after loading. In the test group, at six and nine months, there was no significant change between the mesial and distal surfaces. In the control group, no statistically significant alterations were seen between the mesial and distal surfaces at either six or nine months. Crestal bone level variations across the groups differed significantly (p<0.05) on comparing the data from baseline to six and nine months (Figure 1).

## Discussion

Implantology, a branch of dentistry, has fundamentally altered how patients who are missing a tooth or more may regain their smiles. Implants are deemed to have satisfied the success criterion when they osseointegrate into the host bone bed, support prostheses, and bear occlusal stresses in use [13]. The capacity of the implant to integrate with the surrounding bone and gingival tissue determines how well dental implants function. The amount that the crestal bone level changes and stays steady over time may be used to gauge how well dental implants have worked for a patient. An occlusal overload could be exacerbated by peri-implantitis, which can result from crestal bone loss [14]. Crestal bone resorption would also have an effect on the soft tissues around implants. Based on the position of the bone's crest, Tarnow et al. conducted a study to evaluate if interproximal papilla is present in humans. They discovered that the presence of the apex of the bone's crest is essential for the papilla's health [15]. These elements have increased the importance of maintaining the crestal bone to the implant's functionality.

In order to address several recession defects that were adjacent to one another, Uraz et al. evaluated PRF and connective tissue graft in a clinical setting [16]. PRF was shown to give appropriate wound healing and highly predictable root coverage. Numerous studies have been conducted on PRF research and its therapeutic uses in several branches of dentistry. PRF is used to treat a variety of conditions, including gingival recession, extraction socket healing, cyst enucleation, periodontal abnormalities, ridge preservation grafting, and endodontic operations. The research included 13 individuals, who had 16 implant locations. Until the study's conclusion, all patients had good treatment compliance. At any treated location, there were no side effects found. Mohanty et al. found that the increased workability and ease of manipulation, enhanced tear strength, enhanced clinical healing, and enhanced epithelialization of the lesion on postoperative histological examination make the PRF membrane excellent [17]. Additionally, since PRF is obtained from the patient's own body, there is no danger of allergic reactions and it is inexpensive. Although the study's sample size was somewhat modest, it was in line with the great majority of clinical research on people. Clinical characteristics examined were probing depth, plaque index, bleeding index and Jemt papilla index. At the baseline and six- and nine-month points, every parameter was examined. The pre-surgical phase, when the quality of the bone and the dimensions of the implants to be inserted were chosen, included an evaluation of the CBCT measurements. To examine crestal bone alterations, further radiographic evaluations were performed using the IOPAR paralleling cone method at baseline, six, and nine months.

One of the most important aspects that plays a role in the formation or preservation of peri-implant soft-tissue architecture is the presence of crestal bone. The majority of crestal bone loss happens within the first year of implant function, and it may be as much as 1.2 mm coronoapically. This can be a significant problem. In the present study, crestal bone level variations across the groups differed significantly (p<0.05) when comparing the data from baseline to six and nine months. These findings are in accordance with a previous study done by Boora et al. [10]. While PRF expressed a number of growth factors that support and increase both soft and hard tissue repair, this study's low mean crestal bone level alterations in the PRF group might be explained by this. When implants had to be placed on an uneven bone surface or when the placement of the rough/smooth implant border was not aligned with the lowest bone level of the bone crest, initial crestal bone loss (between baseline and early healing) might also have happened. In addition, it has been shown that the host reacts with an inflammatory response in response to the microgap/interface at the connection to the super-structure, which may have led to tissue remodeling. It has been suggested that bacteria may form a reservoir in such microgaps (interfaces). Therefore, the preservation of crestal bone is a very essential factor that contributes to the success of implant dentistry [10]. In the present study, there was no statistically significant difference in plaque index in both groups between baseline, six and nine months. This was achieved by reinforcing plaque management strategies and recommendations for maintaining good dental hygiene over distinct recall intervals. These findings are consistent with what was shown in earlier research [18,19].

Every patient in our investigation showed signs of a successful recovery after implant installation. The amount of bleeding that happens after inserting a probe into the periodontal pocket is one periodontal criterion for determining whether or not an inflammatory process is present at the base of the pocket. We observed a decrease in the bleeding upon probing between the first and ninth month of our trial. However, the reduction was not statistically significant (p>0.05), which was concordance with earlier studies by Blanes et al., Lekholm et al. and Rismanchian and Fazel [18-20]. Two patients in the test group saw bone outgrowth above the implant's cover screw during the second stage of recovery, demonstrating the additional effect of PRF on the healing process.

Jemt proposed an index to assess the size of the inter-proximal gingival papillae adjacent to single-implant restorations [21]. Our data showed that there was a really significant difference (p<0.05) between the test and control groups at baseline and at the end of six and nine months of the review. The significance difference at baseline is a fascinating finding because it implies that the anatomy of the neighboring tooth, such as its root diameter, the shape of its cementoenamel junction, the attachment of connective tissue, and the amount of mesiodistal space between the implant and tooth, might have a significant impact on the papilla dimensions between the tooth and implant [15]. PRF promotes osteoprogenitor cell proliferation and differentiation [10]. A broad range of human cell types, including trabecular bone cells, osteoblast-like cells, stromal juvenile microorganisms, and human mesenchymal undifferentiated cells, may be stimulated by growth factors derived from platelets [22]. When fibronectin is present, gingival fibroblasts are more likely to stick to and disseminate over the implant surface. Additionally, fibronectin promotes the development of focal adhesions in osteoblasts, allowing remodeling of interdental papilla to occur [23]. Lang et al. showed that in peri-implant health, the connective adaptation area is resistant to probing penetration and will be always present coronal to the interface between the bone and implant, which is in concordance with the present study results, where a significant difference (p<0.05) was seen at baseline between the groups [24]. This discrepancy may have been caused by patient-related elements like the quality of the bone, excessive chewing forces, and the level of oral hygiene of the patient [10]. According to Zhao et al.'s clinical and histological results, filling a fresh extraction socket with PRF offers a possible therapeutic alternative for implant site preparation [25]. In addition, they showed that a histological examination of the core that had been taken out of the socket indicated the development of new bone. Furthermore, the tissue showed no signs of inflammatory infiltrates. According to the clinical and histological evidence, using PRF in a freshly cleaned out extraction socket might be a therapeutic approach for preparing implant sites.

Intraoral periapical x-rays could only provide two-dimensional information. The use of a CBCT scan for three-dimensional bone analysis might have provided more accurate and undistorted findings postoperatively. Additional extensive, long-term studies will be required to verify the contribution of PRF to the bio-functionalization of implants. The two main limitations of this study were that the sample size was quite small and the research did not evaluate cone beam computed tomography.

## Conclusions

We draw the conclusion that the assessment of clinical parameters showed statistically significant differences for probing depth and papilla index score at baseline and six and nine months of loading. No statistically significant difference was present for plaque index and bleeding index at six and nine months of loading. The radiographic assessment showed that the mean crestal bone change over the nine-month period was greater in the control group when compared to the test group. There was a statistically significant difference present between the groups in radiographic assessment evaluating crestal bone changes at baseline, six and nine months of loading. Based on the results of this study, PRF could be viewed as a healing biomaterial with potential beneficial effects on bone formation and can be used as a therapeutic adjuvant in dental implant placement procedures.
